# Supplementary material for: Reconstitution reveals two paths of force transmission through the kinetochore
Source: eLife. 2020 May 14;9:e56582. doi: 10.7554/eLife.56582 (PMC7367685; doi:10.7554/eLife.56582)
Supplement: Figure 1—source data 1. [file elife-56582-fig1-data1.docx]

| Protein complex | Plasmid name | Names used in this paper | Proteins expressed* | Vector | References |
| --- | --- | --- | --- | --- | --- |
| Mif2 | Sc_Mf_7 | Mif2 | Mif2-linker-(27-392)MBP-6XHis** | pLIC | This study |
|  | pGH52 | Mif2 | Mif2-linker-(27-392)MBP** | pLIC | This study |
|  | Sc_Mf_5B | ΔN-Mif2 | (41-549)Mif2-linker-(27-392)MBP | pLIC | This study |
|  |  |  |  |  |  |
| OA | pGH3 | OA | Ame1-6XHis, Okp1 | pST39 | This study |
|  | pGH4 | OA | Ame1-FLAG, Okp1 | pST39 | This study |
|  | pGH42 | ΔN-OA | (21-324)Ame1-FLAG, Okp1 | pST39 | This study |
|  | pGH15 | ΔN-OA | (21-324)Ame1-6XHis, Okp1 | pST39 | This study |
|  |  |  |  |  |  |
| MIND | pGH63 | 2D-MIND | 6XHis-linker-Nsl1, S240D, S250D-Dsn1, Mtw1, Nnf1 | pST39 | This study |
|  | pGH62 | 2D-MIND | FLAG-Nsl1, S240D, S250D-Dsn1, Mtw1, Nnf1 | pST39 | This study |
|  | pGH46 | MIND | Nsl1, FLAG-Dsn1, Mtw1, Nnf1 | pST39 | This study |
|  |  |  |  |  |  |
| Ndc80c | pJT048 | Part of Ndc80c | Spc24-Flag, Spc25 | pRSFDuet | (Kudalkar et al. 2015) |
|  | pEM033 | Part of Ndc80c | Spc24-6XHis, Spc25 | pRSFDuet | (Scarborough, Davis, and Asbury 2019) |
|  | Ndc80/Nuf2 | Part of Ndc80c | Nuf2, Ndc80 | pETDuet | (Wei, Sorger, and Harrison 2005) |
|  |  |  |  |  |  |
| Dam1c | pJT044 | Dam1c | Dad1, Duo1, Spc34-FLAG, Dam1, Hsk3 and  Dad4, Dad3, Dad2, Spc19, Ask1^‡^ | pST39 | (Umbreit et al. 2014) |
|  |  |  |  |  |  |
| CI | pGH58 | CI | FLAG-Chl4, Iml3 | pLIC | This study |
|  |  |  |  |  |  |
| Histones | pScKl2 | Cse4-NCP | *K.lactis* 6XHis-H2A, *K. lactis* 6XHis-H2B, Cse4, *K. lactis* 6XHis-H4 | pLIC | (Migl et al. 2020) |
|  | pScKl4 | H3-NCP | H3, 6XHis-H2A, H2B. *K.lactis* 6XHis-H4 | pLIC | (Migl et al. 2020) |
|  | pScHT4 | Cse4^(1-50)^ | 6XHis-MBP-(1-50)Cse4 | pLIC | This study |

**Figure 1–– source data 1: Plasmids used in this study.**

*Proteins are listed in order of expression in polycistronic vector. C-terminal tags are given on the right side of the protein name and N-terminal tags on the left side of the protein name.

^ Full length Mif2 is expressed by these vectors; the MBP tag includes residues 27-392 of MBP and lacks the signal peptide.

^‡^Dam1 complex is expressed from two polycistrons in one plasmid.
