# Supplementary material for: Reconstitution reveals two paths of force transmission through the kinetochore
Source: eLife. 2020 May 14;9:e56582. doi: 10.7554/eLife.56582 (PMC7367685; doi:10.7554/eLife.56582)
Supplement: Supplementary file 1. — Colour versions of Tables 1 and 2. [file elife-56582-supp1.docx]

Figure 1–– Table Supplement 1. Proteins of the kinetochore.
S. cerevisiae	H. sapiens
Dam1c / DASH	
Ask1 (Associated with spindles and kinetochores)	
Dad1 (Duo1 and Dam1 interacting)	
Dad2 (Duo1 and Dam1 interacting)	Higher eukaryotic analog
Dad3 (Duo1 and Dam1 interacting)	is the Ska complex
Dad4 (Duo1 and Dam1 interacting)	
Dam1 (Duo1 and Mps1 interacting)	
Duo1 (Death upon overproduction)	
Hsk3 (Helper of Ask1)	
Spc19 (Spindle pole component)	
Spc34 (Spindle pole component)	
	
	Ska Complex
Functional analog is Dam1c	Ska1
	Ska2
	Ska3
	
Spc105c	Knl complex
Spc105 (Spindle component)	KNL1
Kre28 (Killer toxin resistant)	Zwint-1
	
Ndc80c	Ndc80c
Ndc80 (Nuclear division cycle)	Hec1
Nuf2 (Nuclear filamentous protein)	Nuf2
Spc24 (Spindle pole component)	Spc24
Spc25 (Spindle pole component)	Spc25
	
MIND	Mis12c
Mtw1 (Mis Twelve-like)	Mis12
Dsn1 (Dosage suppressor of NNF1)	Dsn1
Nnf1 (Necessary for nuclear function)	Pmf1
Nsl1 (NNF1 synthetic lethal)	Nsl1
	
Cnn1c	CENP-TWSX
Cnn1 (Co-purified with Nnf1)	CENP-T
Wip1 (W-like protein)	CENP-W
Mhf1 (Mph1-associated histone-fold protein)	CENP-S
Mhf2 (Mph1-associated histone-fold protein)	CENP-X
	
OA	CENP-QU
Okp1 (Outer kinetochore protein)	CENP-Q
Ame1 (Associated with microtubules and essential)	CENP-U
	
Mif2	CENP-C
Mif2 (Mitotic fidelity of chromosome transmission)	CENP-C
	
CI	CENP-NL
Chl4 (Chromosome loss)	CENP-N
Iml3 (Increased minichromosome loss)	CENP-L
	
NN	
Nkp1 (Non-essential kinetochore protein)	No human homolog
Nkp2 (Non-essential kinetochore protein)	
	
CM	CENP-OP
Ctf19 (Chromosome transmission fidelity)	CENP-O
Mcm21 (Mini-chromosome maintenance)	CENP-P
No fungal homolog	CENP-R
	
CM	CENP-HIKM
Ctf3 (Chromosome transmission fidelity)	CENP-H
Mcm16 (Mini-chromosome maintenance)	CENP-I
Mcm22 (Mini-chromosome maintenance)	CENP-K
No fungal homolog	CENP-M
	
Centromeric histone	Centromeric histone
Cse4 (Chromosome segregation)	CENP-A
S. cerevisiae	H. sapiens
Dam1c / DASH	
Ask1 (Associated with spindles and kinetochores)	
Dad1 (Duo1 and Dam1 interacting)	
Dad2 (Duo1 and Dam1 interacting)	Higher eukaryotic analog
Dad3 (Duo1 and Dam1 interacting)	is the Ska complex
Dad4 (Duo1 and Dam1 interacting)	
Dam1 (Duo1 and Mps1 interacting)	
Duo1 (Death upon overproduction)	
Hsk3 (Helper of Ask1)	
Spc19 (Spindle pole component)	
Spc34 (Spindle pole component)	
	Ska Complex
Functional analog is Dam1c	Ska1
	Ska2
	Ska3
Spc105c	Knl complex
Spc105 (Spindle component)	KNL1
Kre28 (Killer toxin resistant)	Zwint-1
Ndc80c	Ndc80c
Ndc80 (Nuclear division cycle)	Hec1
Nuf2 (Nuclear filamentous protein)	Nuf2
Spc24 (Spindle pole component)	Spc24
Spc25 (Spindle pole component)	Spc25
	
MIND	Mis12c
Mtw1 (Mis Twelve-like)	Mis12
Dsn1 (Dosage suppressor of NNF1)	Dsn1
Nnf1 (Necessary for nuclear function)	Pmf1
Nsl1 (NNF1 synthetic lethal)	Nsl1
	
Cnn1c	CENP-TWSX
Cnn1 (Co-purified with Nnf1)	CENP-T
Wip1 (W-like protein)	CENP-W
Mhf1 (Mph1-associated histone-fold protein)	CENP-S
Mhf2 (Mph1-associated histone-fold protein)	CENP-X
	
OA	CENP-QU
Okp1 (Outer kinetochore protein)	CENP-Q
Ame1 (Associated with microtubules and essential)	CENP-U
	
Mif2	CENP-C
Mif2 (Mitotic fidelity of chromosome transmission)	CENP-C
	
CI	CENP-NL
Chl4 (Chromosome loss)	CENP-N
Iml3 (Increased minichromosome loss)	CENP-L
	
NN	
Nkp1 (Non-essential kinetochore protein)	No human homolog
Nkp2 (Non-essential kinetochore protein)	
	
CM	CENP-OP
Ctf19 (Chromosome transmission fidelity)	CENP-O
Mcm21 (Mini-chromosome maintenance)	CENP-P
No fungal homolog	CENP-R
	
CM	CENP-HIKM
Ctf3 (Chromosome transmission fidelity)	CENP-H
Mcm16 (Mini-chromosome maintenance)	CENP-I
Mcm22 (Mini-chromosome maintenance)	CENP-K
No fungal homolog	CENP-M
	
Centromeric histone	Centromeric histone
Cse4 (Chromosome segregation)	CENP-A
	
	


Protein complex	Plasmid name	Names used in this paper	Proteins expressed*	Vector	References
Mif2	Sc_Mf_7	Mif2	Mif2-linker-(27-392)MBP-6XHis**	pLIC	This study
	pGH52	Mif2	Mif2-linker-(27-392)MBP**	pLIC	This study
	Sc_Mf_5B	ΔN-Mif2	(41-549)Mif2-linker-(27-392)MBP	pLIC	This study
					
OA	pGH3	OA	Ame1-6XHis, Okp1	pST39	This study
	pGH4	OA	Ame1-FLAG, Okp1	pST39	This study
	pGH42	ΔN-OA	(21-324)Ame1-FLAG, Okp1	pST39	This study
	pGH15	ΔN-OA	(21-324)Ame1-6XHis, Okp1	pST39	This study
					
MIND	pGH63	2D-MIND	6XHis-linker-Nsl1, S240D, S250D-Dsn1, Mtw1, Nnf1	pST39	This study
	pGH62	2D-MIND	FLAG-Nsl1, S240D, S250D-Dsn1, Mtw1, Nnf1	pST39	This study
	pGH46	MIND	Nsl1, FLAG-Dsn1, Mtw1, Nnf1	pST39	This study
					
Ndc80c	pJT048	Part of Ndc80c	Spc24-Flag, Spc25	pRSFDuet	(Kudalkar et al. 2015)
	pEM033	Part of Ndc80c	Spc24-6XHis, Spc25	pRSFDuet	(Scarborough, Davis, and Asbury 2019)
	Ndc80/Nuf2	Part of Ndc80c	Nuf2, Ndc80	pETDuet	(Wei, Sorger, and Harrison 2005)
					
Dam1c	pJT044	Dam1c	Dad1, Duo1, Spc34-FLAG, Dam1, Hsk3 and  Dad4, Dad3, Dad2, Spc19, Ask1‡	pST39	(Umbreit et al. 2014)
					
CI	pGH58	CI	FLAG-Chl4, Iml3	pLIC	This study
					
Histones	pScKl2	Cse4-NCP	K.lactis 6XHis-H2A, K. lactis 6XHis-H2B, Cse4, K. lactis 6XHis-H4	pLIC	(Migl et al. 2020)
	pScKl4	H3-NCP	H3, 6XHis-H2A, H2B. K.lactis 6XHis-H4	pLIC	(Migl et al. 2020)
	pScHT4	Cse4(1-50)	6XHis-MBP-(1-50)Cse4	pLIC	This study


Figure 1–– Table Supplement 2:  Plasmids used in this study.  
*Proteins are listed in order of expression in polycistronic vector.  C-terminal tags are given on the right side of the protein name and N-terminal tags on the left side of the protein name.  
^ Full length Mif2 is expressed by these vectors; the MBP tag includes residues 27-392 of MBP and lacks the signal peptide.  
‡Dam1 complex is expressed from two polycistrons in one plasmid. 
