# Supplementary material for: Reconstitution reveals two paths of force transmission through the kinetochore
Source: eLife. 2020 May 14;9:e56582. doi: 10.7554/eLife.56582 (PMC7367685; doi:10.7554/eLife.56582)
Supplement: Transparent reporting form [file elife-56582-transrepform.docx]

***eLife’s* transparent reporting form**

We encourage authors to provide detailed information *within their submission* to facilitate the interpretation and replication of experiments. Authors can upload supporting documentation to indicate the use of appropriate reporting guidelines for health-related research (see [EQUATOR Network](http://www.equator-network.org/%20)), life science research (see the [BioSharing Information Resource](https://biosharing.org/)), or the [ARRIVE guidelines](http://www.plosbiology.org/article/info:doi/10.1371/journal.pbio.1000412) for reporting work involving animal research. Where applicable, authors should refer to any relevant reporting standards documents in this form.

If you have any questions, please consult our Journal Policies and/or contact us: [editorial@elifesciences.org](mailto:editorial@elifesciences.org).

**Sample-size estimation**

- You should state whether an appropriate sample size was computed when the study was being designed
- You should state the statistical method of sample size computation and any required assumptions
- If no explicit power analysis was used, you should describe how you decided what sample (replicate) size (number) to use

Please outline where this information can be found within the submission (e.g., sections or figure legends), or explain why this information doesn’t apply to your submission:

No explicit power analysis was used. For the rupture force assay, I collected data on as many rupture events as I was able to observe in about an hour per slide, on at least three separately prepared slides.

**Replicates**

- You should report how often each experiment was performed
- You should include a definition of biological versus technical replication
- The data obtained should be provided and sufficient information should be provided to indicate the number of independent biological and/or technical replicates
- If you encountered any outliers, you should describe how these were handled
- Criteria for exclusion/inclusion of data should be clearly stated
- High-throughput sequence data should be uploaded before submission, with a private link for reviewers provided (these are available from both GEO and ArrayExpress)

Please outline where this information can be found within the submission (e.g., sections or figure legends), or explain why this information doesn’t apply to your submission:

For every condition for which rupture forces were measured, ruptures were collected from 3-11 separately prepared slides. Beads that did not hold the 1 pN pre-load force were excluded from rupture force analysis; all beads that did withstand the 1 pN pre-load force were included, unless the experiment was interrupted (for instance, by a second bead falling into the trap or by a microtubule polymerizing into another microtubule.) Beads that did not rupture at the maximum force that the trap could exert in a given orientation were included in our analysis as right-censored data. Every individual rupture event is reported in Source Data for Figures 2,3,4, & 5 (and their supplementary figures.)

**Statistical reporting**

- Statistical analysis methods should be described and justified
- Raw data should be presented in figures whenever informative to do so (typically when N per group is less than 10)
- For each experiment, you should identify the statistical tests used, exact values of N, definitions of center, methods of multiple test correction, and dispersion and precision measures (e.g., mean, median, SD, SEM, confidence intervals; and, for the major substantive results, a measure of effect size (e.g., Pearson's r, Cohen's d)
- Report exact p-values wherever possible alongside the summary statistics and 95% confidence intervals. These should be reported for all key questions and not only when the p-value is less than 0.05.

Please outline where this information can be found within the submission (e.g., sections or figure legends), or explain why this information doesn’t apply to your submission:

For the self-assembly and microtubule-binding assay (Figures 2A, 3A, and 5A), Barnard’s Test was used to compare 2x2 contingency tables and calculate p-values. Barnard’s Test was used instead of Fischer’s exact test because it is more powerful. For the rupture force assay (Figures 2B-C, 3B-C, 4A-B, Figure 4 –– figure supplement 1, 5B-c, Figure 5–– figure supplements 1 & 2) a Kolmogorov-Smirnov test was used to compare probability distributions and calculate p-values. This non-parametric statistic was selected because our data are right-censored. Boxplots report medians and quartiles, while whiskers extend to 1.5 times the interquartile range beyond each quartile. These non-parametric statistics are reported because some of our rupture force data are right-censored. All of this information can be found in the corresponding figure legends.

All rupture force data points, n-values, summary statistics, and p-values are included in our Source Data. Because only a small number of planned comparisons were made, each to test a specific hypothesis, p-values have not been adjusted for multiple comparisons.

(For large datasets, or papers with a very large number of statistical tests, you may upload a single table file with tests, Ns, etc., with reference to sections in the manuscript.)

**Group allocation**

- Indicate how samples were allocated into experimental groups (in the case of clinical studies, please specify allocation to treatment method); if randomization was used, please also state if restricted randomization was applied
- Indicate if masking was used during group allocation, data collection and/or data analysis

Please outline where this information can be found within the submission (e.g., sections or figure legends), or explain why this information doesn’t apply to your submission:

N/A.

**Additional data files (“source data”)**

- We encourage you to upload relevant additional data files, such as numerical data that are represented as a graph in a figure, or as a summary table
- Where provided, these should be in the most useful format, and they can be uploaded as “Source data” files linked to a main figure or table
- Include model definition files including the full list of parameters used
- Include code used for data analysis (e.g., R, MatLab)
- Avoid stating that data files are “available upon request”

Please indicate the figures or tables for which source data files have been provided:

Source data have been provided for Figures 2B-C, 3B-C, 4A-B, 5B-C, Figure 4 –– figure supplement 1, Figure 5–– figure supplements 1 & 2.
